# Neuroprotective Effect of Optogenetics Varies With Distance From Channelrhodopsin-2 Expression in an Amyloid-β-Injected Mouse Model of Alzheimer’s Disease

**DOI:** 10.3389/fnins.2020.583628

**Published:** 2020-10-09

**Authors:** Xiaorui Cui, Feng Zhang, Hui Zhang, Xi Huang, Kewei Wang, Ting Huang, Xifei Yang, Liangyu Zou

**Affiliations:** ^1^Department of Neurology, Second Clinical Medical College of Jinan University, Shenzhen People’s Hospital, Shenzhen, China; ^2^Department of Neurology, Affiliated Hospital of Xiangnan University, Chenzhou, China; ^3^Intensive Care Unit, The First Affiliated Hospital, Jinan University, Guangzhou, China; ^4^Department of Neurology, Shenzhen People’s Hospital (First Affiliated Hospital of Southern University of Science and Technology), Second Clinical College, Jinan University, Shenzhen, China; ^5^Department of Neurology, Longgang District People’s Hospital of Shenzhen, Shenzhen, China; ^6^Department of Cerebrovascular Disease, People’s Hospital of Yuxi, The Sixth Affiliated Hospital of Kunming Medical University, Yuxi, China; ^7^Key Laboratory of Modern Toxicology of Shenzhen, Shenzhen Center for Disease Control and Prevention, Shenzhen, China

**Keywords:** Alzheimer’s disease, amyloid-1-42, channelrhodopsin-2, memory, novel object recognition, neuroprotection, neuro-inflammation

## Abstract

**Background:** Alzheimer’s disease (AD) is a progressive neurodegenerative disease that is the most common cause of dementia. Optogenetics uses a combination of genetic engineering and light to activate or inhibit specific neurons in the brain.

**Objective:** The objective of the study was to examine the effect of activation of glutamatergic neurons in the hippocampus of mice injected with Aβ1-42 on memory function and biomarkers of neuroinflammation and neuroprotection in the brain to elucidate the clinical utility of optogenetic neuromodulation in AD.

**Methods:** AAV5–CaMKII–channelrhodopsin-2 (CHR2)–mCherry (Aβ-CHR2 mice) or AAV5—CaMKII–mCherry (Aβ-non-CHR2 mice) was injected into the dentate gyrus (DG) of the bilateral hippocampus of an Aβ1-42-injected mouse model of AD. The novel object recognition test was used to investigate working memory (M1), short-term memory (M2), and long-term memory (M3) after Aβ1-42 injection. Hippocampus tissues were collected for immunohistochemical analysis.

**Results:** Compared to controls, M1 and M2 were significantly higher in Aβ-CHR2 mice, but there was no significant difference in M3; NeuN and synapsin expression were significantly increased in the DG of Aβ-CHR2 mice, but not in CA1, CA3, the subventricular zone (SVZ), or the entorhinal cortex (ENT); GluR2 and IL-10 expressions were significantly increased, and GFAP expression was significantly decreased, in CA1, CA3, the DG, and the SVZ of Aβ-CHR2 mice, but not in the ENT.

**Conclusion:** Activation of glutamatergic neurons by optogenetics in the bilateral DG of an Aβ-injected mouse model of AD improved M1 and M2, but not M3. A single-target optogenetics strategy has spatial limitations; therefore, a multiple targeted optogenetics approach to AD therapy should be explored.

## Introduction

Alzheimer’s disease (AD) is a progressive neurodegenerative disease that is the most common cause of dementia (Aravanis et al., 2007). AD is characterized by pathological changes that include amyloid-β (Aβ) deposition, marked neuronal loss, and tau hyperphosphorylation (Gomez-Isla et al., 1996; Scheff et al., 2006; Crews and Masliah, 2010). Increasingly, evidence suggests that soluble low-molecular-weight Aβ oligomers are associated with neurotoxicity (Lambert et al., 1998; Lesne et al., 2006; Ono et al., 2009). In a novel mouse model, small, soluble Aβ_1–4__2_ oligomers induced extensive neuronal loss *in vivo*, and initiated a cascade of events that mimicked key neuropathological events in AD (Brouillette et al., 2012).

Optogenetics uses a combination of genetic engineering and light to activate or inhibit specific neurons in the brain and explore the functions associated with those neurons (Deisseroth, 2011). Optogenetics has been used to investigate the pathophysiology of Parkinson’s disease and epilepsy, but studies applying optogenetics to AD are scarce.

AAV5–CaMKII–ChR2–mCherry is an adeno-associated virus (AAV) expressing channelrhodopsin-2 (ChR2)–mCherry under the control of the glutametergic neuron promoter, CamKII (Aravanis et al., 2007). The objective of the present study was to examine the effect of activation of glutamatergic neurons in the hippocampus of mice injected with soluble low-molecular-weight Aβ_1–4__2_ on memory function and biomarkers of neuroinflammation and neuroprotection in the brain to elucidate the clinical utility of optogenetic neuromodulation in AD.

## Materials and Methods

### Study Design

All experiments were approved by the Animal Resources Committee, Jinan University, China (No. LL-KT-2011134) and performed according to the Guide for the Care and Use of Laboratory Animals (NIH publication No. 8523, revised 1985).

**FIGURE 1:**
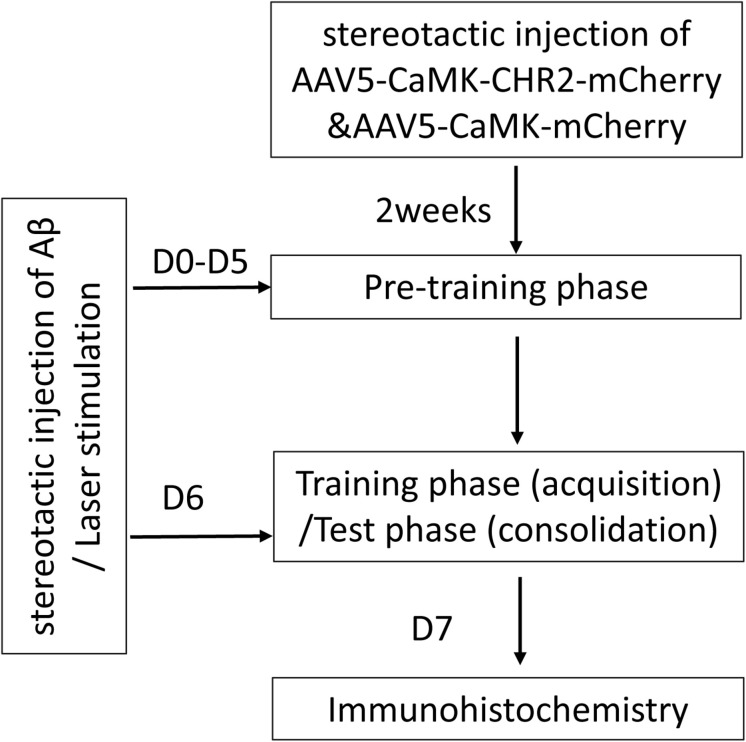
Study design. AAV, adeno-associated virus; CaMK, Ca^2+^/calmodulin-dependent protein kinase; CHR2, channelrhodopsin-2; Aβ, amyloid-β; D, day(s).

A flow chart of the study design is shown in Figure 1. A total of 36 8-month-old female C57BL/6 mice were purchased from Guangdong Medical Laboratory Animal Center, China [license No. SCXK (Yue) 2008-0002]. Mice were housed at 20 ± 2°C and 55 ± 5% humidity, with free access to food and water, under a 12/12 h light/dark cycle. The mice were randomly allocated into three groups: Aβ mice (*n* = 6), Aβ-non-CHR2 mice (*n* = 6), and Aβ-CHR2 mice (*n* = 6). AAV5–CaMKII–CHR2–mCherry (Aβ-CHR2 mice) or AAV5–CaMKII–mCherry (Aβ-non-CHR2 mice) was injected into the dentate gyrus (DG) of the mouse bilateral hippocampus. Fourteen days later, 0.2 μg of soluble low-molecular-weight Aβ_1–4__2_ was injected, and light stimulation with an optical fiber was performed at the same site. Low-molecular-weight Aβ_1–4__2_ injection and light stimulation were repeated once a day for 7 days. Behavioral tests were performed on Day 0 and Days 1–6 after Aβ_1__–__4__2_ injection. Mice were sacrificed on Day 7, and tissues were collected for immunochemical analysis.

### Soluble Low-Molecular-Weight Aβ_1__–__4__2_

Aβ_1__–__4__2_ peptide solution was prepared according to a previously published protocol (Kuperstein et al., 2010; Brouillette et al., 2012). Briefly, Aβ_1__–__4__2_ peptide (Sigma A9810) was dissolved in 99% hexafluoroisopropanol (HFIP) (Sigma-Aldrich) to a concentration of 1 mg/ml. After evaporation under nitrogen gas, the peptide film was dissolved in dimethylsulfoxide (DMSO; Sigma-Aldrich) to a concentration of 1 mg/ml and eluted on a 5 ml HiTrap desalting column (GE Healthcare) with 50 mM Tris, 1 mM EDTA buffer, and pH 7.5. Aβ_1__–__4__2_ concentration was measured with a BCA protein assay kit (Pierce, Rockford, IL, United States). Aβ_1__–__4__2_ was stored on ice and used within 30 min.

### Surgical Procedures

AAV5–CaMKII–CHR2–mCherry and AAV5–CaMKII–mCherry were provided by Shenzhen Institutes of Advanced Technology, Chinese Academy of Sciences. All surgeries were performed under stereotaxic guidance.

Mice were anesthetized with 500 mg/kg of avertin. Bilateral cannulae (328OPD-2.8/Spc with a removable dummy wire; Plastics One) were stereotaxically implanted into the DG of the hippocampus [coordinates with respect to bregma: −2.2 mm anteroposterior (AP), ± 1.4 mm mediolateral (ML), −2.1 mm dorsoventral (DV)], as previously described (Paxinos and Waston, 2005; Brouillette et al., 2012). AAV5–CaMKII–CHR2–mCherry or AAV5–CaMKII–mCherry were injected at 100 nl/min for 10 min to a total of 1 μl through a microelectrode holder (MPH6S;WPI) using a glass micropipette and a 10 μl Hamilton microsyringe (701LT; Hamilton). The needle was retained for 5 min following completion of the injection. Expression of AAV5–CaMKII–CHR2–mCherry and AAV5–CaMKII–mCherry were histologically confirmed 14 days after surgery. Subsequently, Aβ_1__–__4__2_ 0.2 μg/μl was injected into the DG at 100 nl/min for 10 min to a total of 1 μl, as previously described. Next, a fiber optic patchcord optical fiber (200 mm core diameter; Doric Lenses) was implanted at the site of the Aβ_1__–__4__2_ injection, and optical stimulation was generated by a laser (473 nm, 1–3 ms, 10 Hz) (Changchun New Industries) for 5 min.

### Behavioral Test

The novel object recognition test was used to assess the ability of mice to recognize a novel object in their environment. The novel object recognition test was conducted in three phases: (1) Pre-training, mice were allowed to explore an arena without objects for 5 min daily on Day 0 and Days 1–5 after Aβ_1–4__2_ injection. (2) Training phase (acquisition): on Day 6 after Aβ_1__–__4__2_ injection, the mice were placed in the arena with two identical sample objects (A1 and A2) positioned in two adjacent corners 10 cm from the walls. The mice were placed against the center of the opposite wall with their back to the objects. The mice were allowed to explore the objects for 3 min and were then placed in their home cage. A memory index (M0) was calculated as follows: M0 (%) = (exploration time devoted to object A2/exploration time devoted to object A1 + exploration time devoted to object A2) × 100. (3) Test phase (consolidation): mice were placed in the arena with two objects in the same position, one was identical to the sample objects, and the other was novel (A1 and B). The mice were allowed to explore the objects 5 min, 2 h, or 24 h after the training phase to measure working memory (M1), short-term memory (M2), or long-term memory (M3). The memory indices were calculated as follows: M1, M2, M3 (%) = exploration time devoted to object B/(exploration time devoted to object A1 + exploration time devoted to object B) × 100. A higher memory index implied a better ability to recognize a familiar object.

#### Immunohistochemistry

Mouse brain was embedded in paraffin. Brain tissue was sectioned to 30 μm in the coronal plane at the target area and temporarily stored in a 12-well plate in PBS. Sections were treated with xylene and rehydrated in graded ethanol (Fachim et al., 2016). Sections were blocked in 3% BSA at room temperature for 1 h and incubated in 0.3% Triton X-100/PBS with primary antibody overnight at 4°C. Primary antibodies were mouse antiglial fibrillary acidic protein (GFAP, 5 μg/ml, Cat. No. MAB3402, Chemicon), monoclonal mouse anti-NeuN (1:500, Cat. No. MAB377, Millipore), monoclonal mouse anti-synapsin Ia/b (A-1, 1:100, Cat. NO. sc-398849, Santa Cruz), rabbit anti-glutamate receptor 2 (GluR-2, 1:4,000 Cat. No. AB1768, Millipore), or mouse anti-interleukin (IL)-10 (A-2, 1:100 Cat. No. sc-365858, Santa Cruz). After washing, sections were incubated with secondary antibody in the dark for 1 h at room temperature. Secondary antibodies were goat anti-mouse IgG (H&L, 1:2,000 Cat. No. ab7067; Abcam) or goat anti-rabbit IgG (H&L, HRP, 1:2,000 Cat. No. ab6721; Abcam). Images of CA1, CA3, the DG, the subventricular zone (SVZ), and the entorhinal cortex (ENT) were visualized with a light microscope (DMI 3000 B; Leica, Buffalo Grove, IL, United States). The number of immunostained-positive cells was counted using Image J software (NIH, Bethesda, MD, United States) in a double-blind manner and was expressed as a percentage of the Aβ mice.

### Statistical Analysis

Statistical analyses were performed using SPSS19.0 and Prism 6 (GraphPad). Data are presented as mean ± SEM. Data from the behavioral tests were compared using repeated measures analysis of variance. Data from immunohistochemical analysis were compared with one-way analysis of variance. *P* < 0.05 was considered statistically significant.

## Results

### Effect of AAV5–CaMK–CHR2–mCherry on Memory Function in Mice

**FIGURE 2:**
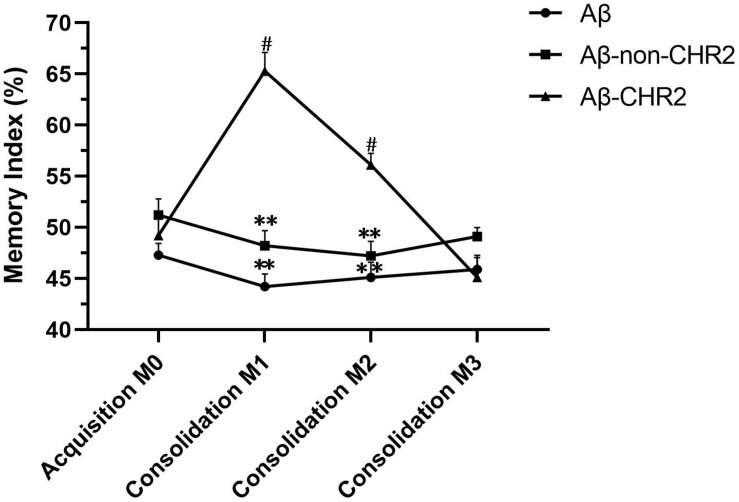
Novel object recognition test. Working, short-term, and long-term memory were assessed based on the memory index during the test phase (consolidation), 5 min, 2 h, and 24 h after the training phase. Data are expressed as the mean ± SEM (*n* = 8). **P < 0.001, vs. the Aβ-CHR2 group. Aβ mice received repeated injections of A_β1–42_ in the bilateral DG; Aβ-non-CHR2 mice received AAV5–CaMKII–mCherry and repeated injections of Aβ_1–42_ in the bilateral DG, as well as light stimulation; Aβ-CHR2 mice received AAV5–CaMKII–CHR2–mCherry and repeated injections of Aβ_1–42_ in the bilateral DG as well as light stimulation. Memory index (%) = exploration time devoted to object B/(exploration time devoted to object A1 + exploration time devoted to object B) × 100. AAV, adeno-associated virus; CaMK, Ca^2+^/calmodulin-dependent protein kinase; CHR2, channelrhodopsin-2. ^#^*P* < 0.001, vs. M0 (repeated measures analysis of variance).

M1 and M2 were significantly increased compared to M0 in Aβ-CHR2 mice (*F* = 25.12, *P* < 0.0001), but there was no significant difference between M0 and M3 (*P* > 0.05). There were no significant differences between M0, M1, M2, and M3 in Aβ-non-CHR2 mice and Aβ mice (Aβ-non-CHR2 mice, *F* = 1.524, *P* > 0.05; Aβ mice, *F* = 1.099, *P* > 0.05). M1 and M2 were significantly higher in Aβ-CHR2 mice compared to Aβ-non-CHR2 mice and Aβ mice (*F* = 53.93, *P* < 0.001 for M1; *F* = 18.31, *P* < 0.001 for M2). There were no significant differences in M3 in Aβ-CHR2 mice, Aβ-non-CHR2 mice, and Aβ mice (*F* = 2.002, *P* > 0.05) (Figure 2). These results suggest that working memory and short-term memory, but not long-term memory, were rescued by optogenetic treatment.

### Effect of AAV5–CaMKII–CHR2–mCherry on NeuN and Synapsin Expression in CA1, CA3, the DG, the SVZ, and the ENT

**FIGURE 3:**
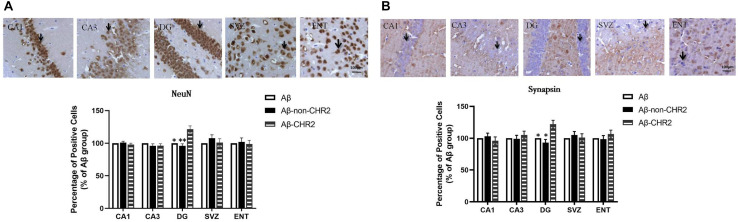
Effect of CHR2 on NeuN **(A)** and synapsin **(B)** expression in CA1, CA3, the DG, the SVZ and the ENT (*n* = 6). Arrows show the positive cells, 400×. ^∗^*P* < 0.05, ^∗∗^*P* < 0.01 vs. the Aβ-CHR2 mice. Aβ mice received repeated injections of Aβ_1__–__4__2_ in the bilateral DG; Aβ-non-CHR2 mice received AAV5–CaMKII–mCherry and repeated injections of Aβ_1__–__4__2_ in the bilateral DG, as well as light stimulation; Aβ-CHR2 mice received AAV5–CaMKII–CHR2-mCherry and repeated injections of Aβ_1__–__4__2_ in the bilateral DG as well as light stimulation. DG, dentate gyrus; ENT, entorhinal cortex; NeuN, neuronal nuclei; SVZ, subventricular zone.

NeuN and synapsin expressions were significantly increased in the DG of Aβ-CHR2 mice compared to that of Aβ-non-CHR2 mice and Aβ mice (*P* < 0.05). There were no significant differences in NeuN and synapsin expression in CA1, CA3, the SVZ, or the ENT of Aβ-CHR2 mice, Aβ-non-CHR2 mice, and Aβ mice (*P* > 0.05) (Figure 3).

### Effect of AAV5–CaMKII–CHR2–mCherry on GluR2, IL-10, and GFAP Expression in CA1, CA3, the DG, the SVZ, and the ENT

**FIGURE 4:**
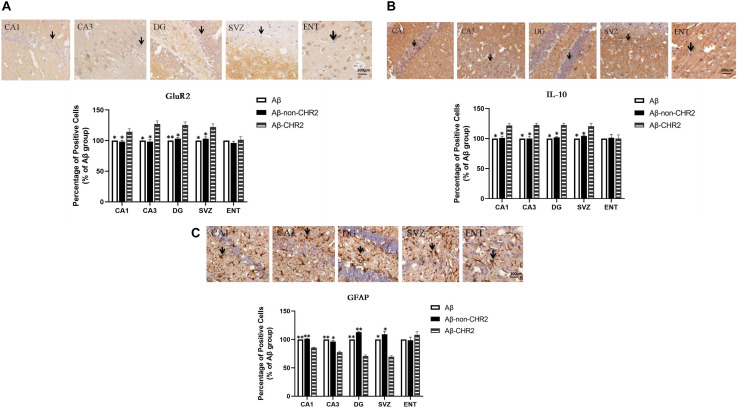
Effect of CHR2 on GluR2 **(A)**, IL-10 **(B)**, and GFAP **(C)**, expression in CA1, CA3, DG, the SVZ, and the ENT (*n* = 6). Arrows show positive cells, 400×. *P < 0.05, **P < 0.01 vs. Aβ-CHR2 mice. Aβ mice received repeated injections of Aβ_1__–__4__2_ in the bilateral DG; Aβ-non-CHR2 mice received AAV5–CaMKII–mCherry and repeated injections of Aβ_1–4__2_ in the bilateral DG, as well as light stimulation; Aβ-CHR2 mice received AAV5–CaMKII–CHR2–mCherry and repeated injections of Aβ_1–4__2_ in the bilateral DG as well as light stimulation. DG, dentate gyrus; ENT, entorhinal cortex; GFAP, glial fibrillary acidic protein; GluR2, glutamate receptors; IL, interleukin; NeuN, neuronal nuclei; SVZ, subventricular zone.

GluR2 and IL-10 expressions were significantly increased, and GFAP expression was significantly decreased in CA1, CA3, the DG, and the SVZ of Aβ-CHR2 mice compared to Aβ-non-CHR2 mice and Aβ mice (*P* < 0.05). There were no significant differences in GluR2, IL-10, and GFAP expression in the ENT of Aβ-CHR2 mice, Aβ-non-CHR2 mice, and Aβ mice (*P* > 0.05) (Figure 4).

## Discussion

This study used optogenetics and investigated the effect of stimulating CaMK–CHR2-expressing neurons in the DG of the bilateral hippocampus on memory function and biomarkers of neuroinflammation and neuroprotection in the brain of an Aβ-injected mouse model of AD. Findings showed: (1) optogenetics improved working memory and short-term memory, but not long-term memory, in Aβ-CHR2 mice, and (2) optogenetics activated GluR2, attenuated neuroinflammation, and exerted neuroprotective effects in the core but not the peripheral areas of CHR2 expression.

Optogenetics enables precise temporal control of neuronal activity and has been used in a number of contexts (Gradinaru et al., 2009; Tye et al., 2011). Bi et al. (2006) proposed the expression of microbial-type channelrhodopsins, such as ChR2, in surviving inner retinal neurons as a potential strategy for the restoration of vision after rod and cone degeneration. Van den Oever et al. (2013) used optogenetics to explore the involvement of ventromedial prefrontal cortex (vmPFC) pyramidal cells in recent and remote conditioned cocaine memory in mice. Activation of pyramidal cells resulted in the loss of remote memory, without affecting recent memory, and inhibition of pyramidal cells impaired recall of recent memory, without affecting remote memory (Van den Oever et al., 2013).

Cognitive impairment in AD is characterized by memory disorders, mental and behavioral changes, insomnia, and autonomic dysfunction (Greene et al., 1996). Memory is a complex phenomenon, and memory impairment is the most prominent symptom of AD. In the present study, a novel object recognition test was used to assess memory function in an Aβ-injected mouse model of AD. The novel object recognition test has been used to evaluate the ability of mice to recognize a novel object in familiar surroundings (Ennaceur and Delacour, 1988) and to recognize an object after administration of bilateral lidocaine (Hammond et al., 2004), providing information on working memory, short-term memory, and long-term memory. The present study demonstrated that M1 and M2 were significantly higher in Aβ-CHR2 mice compared to Aβ-non-CHR2 mice and Aβ mice, and there were no significant differences in M3 in Aβ-CHR2 mice, Aβ-non-CHR2 mice, and Aβ mice. This implies that optogenetics improved working memory and short-term memory, but not long-term memory, in Aβ-CHR2 mice.

The hippocampus is severely affected early in the AD process (Hyman et al., 1984; Hyman et al., 1994). The hippocampus proper, which is defined by CA1–CA3 and the dentate gyrus, is the core structure within a larger hippocampal formation, which includes the adjacent subicular and rhinal cortices. The entorhinal cortex is among the first of the medial temporal lobe regions to exhibit dysfunction in early AD (Khan et al., 2014). Therefore, the neurobiological mechanisms underlying the improvement in memory function after optogenetic activation in the Aβ-injected mouse model of AD were investigated using histological studies of the neurons and synapses in the mouse hippocampus and entorhinal cortex.

NeuN and synapsin have neuroprotective effects. NeuN is a biomarker for arcuate neurons, and synapsins are involved in synaptogenesis and plasticity of mature synapses and play a major role in maintaining brain physiology (Meunier et al., 2015). Synapsins I and II are the major synapsin isoforms in neurons; both can be recognized by anti-synapsin Ia/b. Synapsin I is associated with elongation of axons and regulation of synaptic vesicle fusion. Synapsin II is essential for the synaptic vesicle cycle through its involvement in vesicle docking (Mirza and Zahid, 2018). In the present study, NeuN and synapsin expression in the core area of CHR2 injection was significantly increased, while there was no difference in NeuN and synapsin expression in the peripheral areas of CHR2 expression, including CA1, CA3, the SVZ, and the more distant ENT, compared to controls. This suggests that optogenetic activation of glutamatergic neurons in the DG exerted neuroprotective effects locally, but the effects of optogenetics declined or disappeared with distance from CHR2 expression.

Various regions of the brain are involved in executive memory. The medial temporal lobe (hippocampal system), prefrontal cortex, diencephalon (papillary body and thalamus), and amygdala are reciprocally connected and associated with learning and memory (Naya et al., 2017; Shirayama et al., 2017; Guo et al., 2019). Short-term memory (including working memory) and long-term memory are separate systems. The neural basis of short-term memory and long-term memory are located in the hippocampus and multiple cortical regions, respectively (Matthews, 2015; Hampson et al., 2018). In the present study, the neuroprotective effect of optogenetics was limited to the DG and may have been one mechanism underlying the observed improvement in working memory and short-term memory in Aβ-CHR2 mice. As optogenetic activation of neurons in the DG did not extend to the cortex, there was no obvious enhancement of long-term memory.

Optogenetics combines optics and genetics to control well-defined events in tissues or behaviors in animals (Duebel et al., 2015). It drives physiological changes in a tissue by influencing neurons or synapses via cytokines or neurotransmitters (Van den Oever et al., 2013). Aβ is a pathological hallmark of AD, and Aβ-injected mouse models of AD show AD-like behavioral abnormalities and Aβ pathology. Here, optogenetics was used to activate glutamatergic neurons in the brain of an Aβ-injected mouse model of AD.

The glutamate family of receptors includes the ionotropic receptors [e.g., α-amino-3-hydroxy-5-methyl-4-isoaxolepropionate (AMPA)] and metabotropic receptors (mGluR; G-protein coupled). AMPA receptors are comprised of different combinations of GluR1–GluR4 subunits. RNA editing at the Q/R site of the GluR2 subunit confers Ca^2+^ impermeability to AMPA receptors. The edited form represents nearly 100% of GluR2 subunits expressed in the adult mammals’ brain (Burnashev et al., 1992; Borges and Dingledine, 1998). Thus, the presence of the edited GluR2 subunit plays a key role in determining a neuron’s vulnerability to glutamate toxicity (Palmer and Gershon, 1990). In the present study, optogenetics increased GluR2 expression in CA1, CA3, the DG, and the SVZ, but not in the ENT.

Glutamate is the most abundant free amino acid in the brain and is the major excitatory neurotransmitter in the mammalian central nervous system (Meldrum, 2000; Reiner and Levitz, 2018). Evidence suggests that AD is characterized by impaired glutamate uptake, alterations in the glutamate–glutamine cycle (Walton and Dodd, 2007), and glutamatergic excitotoxicity (Palmer and Gershon, 1990; Lau and Tymianski, 2010), whereby the neurotoxic action of glutamate follows the overactivation of Ca^2+^ -permeable ionotropic glutamate receptors (Choi, 1992).

The maintenance of normal glutamatergic neurotransmission and glutamate clearance depends on active glutamate uptake into glial cells and neurons as glutamate released by neuronal cells is not subsequently metabolized in the extracellular space (Malik and Willnow, 2019). Excitatory amino acid transporters (EAATs) are needed to maintain a low glutamate concentration in the extracellular space and prevent excitotoxicity (Logan and Snyder, 1971; Tanaka et al., 1997). Activation of mGluR2/3 increases the levels of EAAT1 and 2 proteins (Aronica et al., 2003; Lyon et al., 2008; Lin et al., 2014), and mice deficient in mGluR2 have decreased levels of EAAT3 mRNA (Lyon et al., 2008). In the present study, an increase in GluR2 may have upregulated the expression of the EAATs, causing bulk glutamate uptake from the extracellular space and preventing excitotoxicity. This may be one mechanism by which optogenetics with CaMKII targeting glutamatergic neurons exerts a neuroprotective effect.

Findings regarding the associations between AD and inflammatory cytokines, including interleukin (IL)-1β, IL-2, IL-4, IL-6, IL-8, IL-12, IL-18, tumor necrosis factor (TNF)-α, transforming growth factor (TGF)-β, interferon (IFN)-γ, and the C-reactive protein are controversial (Julian et al., 2015). However, IL-10, a cytokine with anti-inflammatory properties, may be a main cytokine associated with the pathogenesis of AD (Swardfager et al., 2010; Sardi et al., 2011; Kiyota et al., 2012). IL-10 limits the immune response to pathogens and microbial flora. AAV serotype 2/1 hybrid-mediated neuronal expression of the mouse IL-10 gene in hippocampal neurons of amyloid precursor protein + presenilin-1 bigenic mice resulted in sustained expression of IL-10, reduced astro/microgliosis, enhanced plasma Aβ levels, and enhanced neurogenesis.

Glial fibrillary acidic protein (GFAP) is a commonly used marker for astrocytes (Sofroniew and Vinters, 2010). Aβ increases GFAP levels in the hippocampus (Meunier et al., 2015), and GFAP is upregulated in astrocytes of patients with AD (Perez-Nievas and Serrano-Pozo, 2018), which initiates neuroinflammation and cellular damage. AAV vectors containing the astrocyte-specific Gfa2 promoter to target hippocampal astrocytes and interfere with the biochemical cascades leading to astrocyte activation in APP/PS1 mice confirmed a deleterious role for activated astrocytes in AD.

In the present study, increased GluR2 expression may have alleviated excitotoxicity, upregulated IL-10, and downregulated GFAP. Thus, diminished neuroinflammation induced by optogenetics may have protected neurons and synapses from the neurotoxicity of Aβ. It was noteworthy that there was increased expression of glutamate receptors (GluR2) and IL-10 and decreased expression of GFAP in CA1, CA3, the DG, and the SVZ, but not the ENT, which is distant to the injection site. Neuroprotection induced by optogenetics was limited to the core area of AAV5–CaMKII–CHR2–mCherry injection. In addition, activation of glutamatergic neurons by AAV5–CaMKII–CHR2–mCherry injection increased NeuN and synapsin expression in the core area (DG) of CHR2 injection, while there were significant changes in the expression of GluR2, IL-10, and GFAP in the core and peripheral areas of CHR2 expression, including CA1, CA3, and the SVZ. This suggests that optogenetic activation of glutamatergic neurons with the CaMKII–CHR2 gene has an extensive effect on astrocytes, although the interaction and mechanism need to be investigated in future studies.

The neuronal–glial network is a potential target for intervention in AD. Consistent with this, our optogenetic technique that selectively stimulated CaMKII–CHR2-expressing neurons in the DG of the bilateral hippocampus improved working memory and short-term memory, altered neuroinflammation, attenuated excitotoxicity induced by Aβ, and exerted neuroprotective effects in our mouse model of AD. This effect was likely mediated by the neuronal–glial network and activation of glutamate receptors.

While optogenetics has temporal precision, spatial resolution, and neuronal specificity, it has inevitable limitations. In the present study, increased NeuN and synapsin expression were only found in the DG, and increased IL-10 and GluR2 expression and decreased GFAP expression were not found in the ENT of the Aβ-injected mouse model of AD. This implies that activation of glutamatergic neurons in the DG modulated neuroinflammation in local and peripheral areas and exerted neuroprotective effects locally, and the effects of optogenetics varied with the distance from CHR2 expression.

Thus, although optogenetics has a potential as an effective treatment for AD, a single-target strategy has spatial limitations. AD has a wide range of injuries, and a multiple targeted optogenetics approach may be a more effective therapy.

## Conclusion

In conclusion, activation of glutamatergic neurons by optogenetics in the bilateral DG of an Aβ-injected mouse model of AD improved working memory and short-term memory and downregulated biomarkers of neuroinflammation in the core and peripheral areas of CHR2 expression and upregulated biomarkers of neuroprotection in the core area of CHR2 expression. Due to the spatial constraints of optogenetics, a multiple targeted approach may be needed to address the heterogeneous clinical presentation and pathology of AD.

## Data Availability Statement

The raw data supporting the conclusions of this article will be made available by the authors, without undue reservation.

## Ethics Statement

All experiments were performed following approval by the Jinan University Animal Resources Committee and according to recommended standards for the care and use of laboratory animals.

## Author Contributions

LZ and XY contributed to the conception and design of the study. XC, FZ, and HZ performed the novel object recognition test and immunohistochemical analyses. XC, FZ, and LZ performed the statistical analysis. XC wrote the first draft of the manuscript. XH, KW, and TH wrote sections of the manuscript. All authors contributed to manuscript revision, and read and approved the submitted version.

## Conflict of Interest

The authors declare that the research was conducted in the absence of any commercial or financial relationships that could be construed as a potential conflict of interest.

## Abbreviations

AD: Alzheimer’s disease
DG: dentate gyrus
ENT: entorhinal cortex
GFAP: glial fibrillary acidic protein
GluR2: glutamate receptors
IL: interleukin
NeuN: neuronal nuclei
SVZ: subventricular zone.
